# Integrative Identification of Candidate Protein Targets and Compounds for Dystonia Using Mendelian Randomization, Single-Cell RNA Sequencing, and Network Pharmacology

**DOI:** 10.3390/genes17091067

**Published:** 2026-09-03

**Authors:** Lin Chen, Ming-Juan Fang, Nan Cheng, Yin Xu

**Affiliations:** 1 Anhui University of Chinese Medicine, Hefei 230061, China; chenlin77990802@163.com; 2 Institute of Neurology, Anhui University of Chinese Medicine, Hefei 230061, China; fangmj99@163.com

**Keywords:** dystonia, drug target, proteome-wide mendelian randomization, single-cell RNA, network pharmacology

## Abstract

Background: Dystonia is a severe neurological disorder with enigmatic pathogenesis. Current treatment options are limited in preventing the disease progression, underscoring the urgent need for new targeted therapeutic agents to develop more effective therapies. Methods: We performed a proteome-wide Mendelian randomization (MR) study and sensitivity analyses to evaluate the causal relationships between dystonia and proteins. GO and KEGG enrichment analysis of dystonia-associated proteins was conducted. Then, we built PPI network and identified the expression of hub-genes in specific brain neurons in single-cell sequencing data. Additionally, we performed drug enrichment analysis of hub-genes, and employed network pharmacology and molecular docking methods to identify potential drugs for dystonia. Results: Our study identified genetically predicted associations consistent with a potential causal effect between 51 proteins and risk of dystonia. GO and KEGG enrichment analyses revealed that these proteins are involved cellular response to transforming growth factor-β stimulation and cytokine-cytokine receptor interaction. Notably, the PPI network exhibited 21 community relationships within the regulatory network among the 51 dystonia-associated proteins identified. The single-cell RNA annotations for brain cluster specificity revealed Tumor necrosis factor (TNF) was highly expressed in microglia cells. Drug enrichment analysis identified five traditional Chinese medicine monomers (paeoniflorin, artesunate, ginsenoside Rh1, psoralen, and quercetin dihydrate) as candidates for molecular docking analysis. Among these, paeoniflorin-TNF, quercetin dihydrate-TNF, and artesunate-TNF exhibited the highest binding energy (−9.1 kcal/mol). Conclusions: Our molecular-docking analysis suggested that traditional Chinese medicine monomers including paeoniflorin, quercetin dihydrate, and artesunate may serve as promising candidates for future drug development.

## 1. Introduction

Dystonia is a disorder of nervous system characterized by involuntary muscle contractions, leading to aberrant movements and atypical postures. As a heterogeneous cluster of extrapyramidal disorders, dystonia ranks third in prevalence among movement disorders following Parkinson’s disease and essential tremor [1]. However, it should be noted that the complexity of the pathophysiology of dystonia poses a challenge for research and therapeutic development [2,3]. Currently, the pharmacological management of dystonia primarily includes the use of anticholinergic drugs, benzodiazepines, muscle relaxants, and levodopa. For the treatment of dystonia, most drugs are ineffective, and the success rate of new drug discovery is in constant decline.

Exploring specific and effective treatments for dystonia has always been a hot topic update. Although conventional medication was used clinically, the treatment efficacy is suboptimal, and some adverse drug reactions have been observed [4]. The fact that numerous approved or experimental drugs target the biological efficacy of many circulating proteins encoded by druggable genes in serum or plasma, highlights their significance as potential therapeutic targets [5]. Screening precise target proteins and providing corresponding treatments, is eagerly awaited. Recently, Mendelian randomization (MR) analysis has become a prevalent tool for uncovering new therapeutic targets and repurposing licensed drugs [6,7]. Genome-wide association studies (GWAS) have the advantage to identify specific single nucleotide polymorphisms (SNPs) located on chromosomes that govern protein expression. The SNPs demonstrate a direct correlation with quantitative traits associated with protein abundance, commonly referred to as protein quantitative trait loci (pQTL) [8]. The utilization of pQTLs as instrumental variables (IVs) in MR analysis offers significant power for investigating potential causal relationships between exposure (protein) and outcomes (disease), thereby facilitating the identification of drug targets and biomarkers for disease. As far as we know, currently, few studies have been conducted on the treatment of dystonia with traditional Chinese medicine (TCM) monomers.

Network pharmacology (NP) is an innovative discipline that employs network-based methodologies to elucidate the complex interconnections among TCM, bioactive compounds, therapeutic targets, signaling pathways, and diseases. The emergence of cutting-edge and intelligent technologies, including proteomics, transcriptomics, single-cell omics, and artificial intelligence, has not only refined NP but also accelerated its in-depth application [9]. As a revolutionary drug-discovery paradigm, NP focuses on uncovering and addressing the root causes of diseases rather than merely alleviating symptoms. Additionally, TCM monomers demonstrate remarkable efficacy in disease treatment. Their well-defined chemical compositions enable precise targeting of disease-related pathways, thus promoting in-depth investigations for rational clinical applications. Standardized manufacturing processes ensure consistent quality and predictable therapeutic outcomes. Furthermore, their single-component structure simplifies adverse reaction monitoring, reduces the risk of drug interactions. In the drug discovery process, molecular docking technology can be employed for the virtual screening of extensive compound libraries, efficiently identifying potential drug molecules with high affinity for disease-related targets.

Therefore, we first conduct a large-sample GWAS to investigate the causal relationship between proteomics and diseases. Subsequently, we integrated several bioinformatics methodologies, including single-cell RNA sequencing, network pharmacology analysis, and molecular docking simulations, to identify potential TCM monomers for targeted therapeutic approaches in dystonia. Firstly, a two-sample Proteome-wide MR analysis was performed to determine the causal effect of plasma proteins on dystonia, and performed GO and KEGG enrichment analysis. Subsequently, we built PPI network and identified hub-genes using Cytoscape (3.10.4) and further examined the expression of these hub-genes in specific brain neurons through the analysis of single-cell sequencing data. Additionally, we performed drug enrichment and employed network pharmacology and molecular docking methods to identify potential TCM monomers for the treatment of dystonia. Our study may contribute to providing novel insights into targeted treatment strategies for dystonia.

## 2. Materials and Methods

### 2.1. Study Design

**Figure 1:**
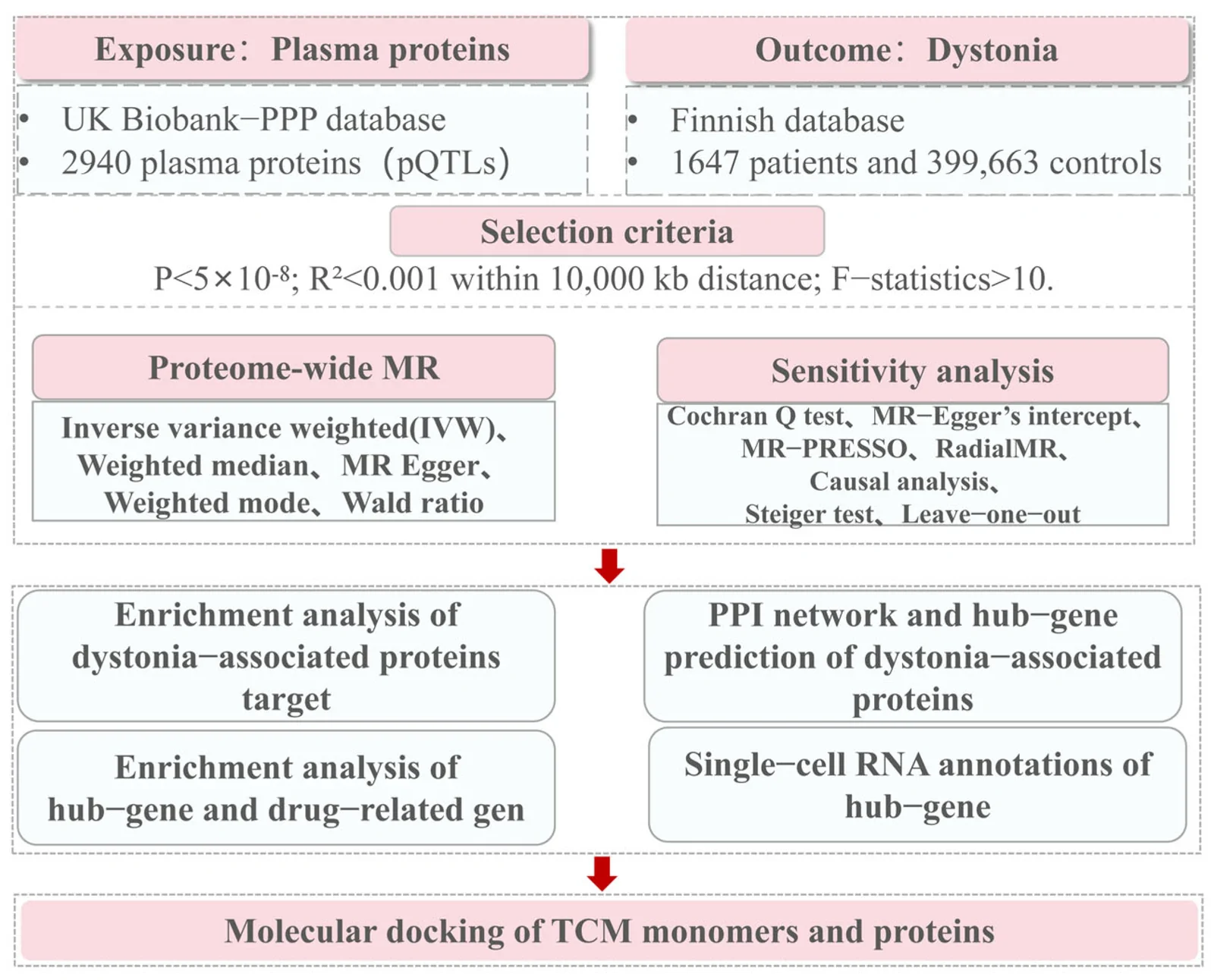
**Flowchart of the study design.** The summary-level data of the pQTLs and dystonia utilized in the present study were originated from publicly available European-descent GWAS. Proteome-wide MR study, single-cell RNA, network pharmacology, and molecular docking was used to systematically screen for potential protein biomarkers and identify TCM monomers target for dystonia. *pQTLs: protein quantitative trait loci; TCM: traditional Chinese medicine; GWAS: Genome-wide association studies.*

The study design is shown in Figure 1. We conducted a proteome-wide MR study, single-cell RNA, network pharmacology, and molecular docking to systematically screen for potential protein biomarkers and identify TCM monomers target for dystonia. The summary-level data of the pQTLs and dystonia utilized in the present study were originated from publicly available European-descent GWAS. The protocol and data collection were approved by the ethics committee of the original GWAS, and written informed consents were obtained from each participant before data collection.

### 2.2. GWAS Data of Plasma pQTLs and Dystonia

Plasma pQTLs were obtained from the plasma proteomic profiles of 54,219 participants enrolled in the UK Biobank-PPP database, sourced from the UK Biobank (Sun et al. External, opens in a new tab. https://metabolomips.org/ukbbpgwas/, accessed on 29 August 2026). A comprehensive mapping was performed to identify pQTLs associated with 2923 proteins [10]. In our proteome-wide MR study focused on drug targets, we deliberately employed cis-pQTLs (not trans-PQTL) as IVs, adhering to specific criteria for selection. The criteria are delineated as follows: (I) pQTLs must reach the threshold of genome-wide significance (*p* < 5 × 10^−8^); (II) The pQTLs should manifest minimal linkage disequilibrium (LD clumping r2  <  0.001) to ensure no significant associations; (III) only cis-acting variants are considered; and (IV) the *F*-statistic for each protein’s pQTL should exceed 10 in order to alleviate bias caused by weak IVs [5,11]. The data for dystonia genetic association research is derived from the R10 version of the Finnish database (https://r10.finngen.fi/, accessed on 29 August 2026). The diagnostic criteria of dystonia are set by the ICD-10 code G24, in conjunction with the corresponding codes 3336 from ICD-9 and 33,100 from ICD-8 (https://risteys.finregistry.fi/endpoints/G6_DYSTON, accessed on 29 August 2026). The dataset consists of data from 1647 patients and 399,663 controls. This endpoint specifically captures primary (idiopathic) dystonia (ICD-10 G24, excluding G24.0 drug-induced dystonia and secondary dystonia due to identifiable neurological conditions). The included subtypes encompass focal dystonia (including cervical dystonia, blepharospasm, writer’s cramp), segmental dystonia, and generalized dystonia.

### 2.3. Mendelian Randomization Analysis and Sensitivity Analysis

In our study, we managed proteins (pQTLs) as exposures and dystonia as outcomes. We utilized the R package “TwoSampleMR” (V.0.6.0.) for conducting MR analysis. A total of five methods were employed for conducting MR analysis, including the Wald ratio method, inverse-variance weighting (IVW), MR Egger, weighted median, and weighted model to estimate causality [12,13]. The statistical significance was determined by a *p*-value below 0.05. Odds ratios (OR) for an increased risk of dystonia were presented per standard deviation (SD) increment in plasma protein levels. We used 6 methods for sensitivity test to confirm most robust results. Firstly, it is imperative to investigate the presence of heterogeneity and employ the Cochran Q test for assessing the statistical significance of genetic variation heterogeneity. In cases where the obtained *p*-value exceeds 0.05, it can be inferred that there is no significant evidence of heterogeneity [13]. Secondly, to test horizontal pleiotropy, we performed two methods including MR-Egger’s intercept [14] and Causal Analysis Using Summary Effect estimates (CAUSE) [15], with *p* > 0.05 showing no horizontal pleiotropy. Thirdly, we used MR Pleiotropy Residual Sum and Outlier (MR-PRESSO) method to identify outliers. A *p*-value of MR-PRESSO less than 0.05 for the Global test indicated the presence of horizontal pleiotropic outliers [16]. We further used Radial MR to test influential outlier IVs [17]. We applied Steiger filtering methodology to test and verify the direction of associations between identified proteins and dystonia [18]. To control for multiple testing across 941 plasma proteins, we applied Benjamini-Hochberg FDR correction (*q* < 0.05).

### 2.4. Functional and Pathway Enrichment Analysis

We performed functional and pathway enrichment analyses on the genes mapped to the proteins identified through MR. The “clusterProfiler” and “enrichplot” R package was employed for enrichment analysis of Gene Ontology (GO) and KEGG pathways, GO including Biological Processes (BP), Cellular Components (CC), Molecular Functions (MF) [19]. We set the significance threshold at FDR < 0.05, and the top 10 most significant GO terms and pathways were visualized using the “ggplot2” R package.

### 2.5. Protein-Protein Interaction Network Analysis

After identifying a multitude of causally related proteins by MR, we performed an extensive Protein-Protein Interaction (PPI) analysis to investigate both direct and indirect interactions among the identified proteins, as well as to validate their functional significance in the pathogenesis of dystonia. We marked regulatory network among dystonia-related proteins to identify the communities formed by the densely connected nodes in the network. Bioinformatics tools and databases, such as STRING (https://string-db.org/, accessed on 29 August 2026), Cytoscape were used to systematically construct and analyze the PPI networks of the relevant proteins. Utilize the “cytoHubba” plugin to identify hub-genes within the Cytoscape.

### 2.6. Single-Cell Transcriptomic Profiling

Given that dystonia is a neurological movement disorder characterized by involuntary muscle contractions, our research specifically targeted brain as selected tissue. We gained the single-cell RNA-sequencing (scRNA-seq) data of brain tissue from the Panglao DB database (https://https://panglaodb.se/, accessed on 29 August 2026), a user-centric single-cell sequencing dataset for the scientific community that focused on single-cell RNA sequencing tests from mice and humans [20]. To verify the stability of the results, we systematically selected two representative datasets for mutual verification. We utilized the “Sample” module to search two datasets. “Human” and “Tissue” were set as screening conditions. The two databases encompassed 6689 and 4339 single-cell RNA-sequencing dataset samples, separately. The publicly attainable datasets utilized in the present study had acquired the necessary ethical approvals.panglaodb.se/2930.

### 2.7. Enrichment-Based Drug Screening and Molecular Docking Validation

We conduct drug enrichment analysis on the hub-genes of the network to identify which drugs are significantly associated with these genes. Drug selection followed a five-step prioritization pipeline: (1) DSigDB enrichment (False Discovery Rate, FDR < 0.05); (2) restriction to TCM-derived small molecules; (3) focus on TNF-targeting compounds; (4) binding energy threshold < −7.0 kcal/mol; (5) multi-criteria evaluation incorporating anti-neuroinflammatory evidence, BBB permeability prediction, safety profile, bioavailability, and drug-likeness. For this purpose, utilize the DSigDB website (version 1.0) by downloading the “DSigDBv1.0 Detailed.txt” file. Extract the target genes for all drugs listed in the database and generate corresponding drug-target gene files for further analysis using R. We obtained the three-dimensional structures of each small molecule ligand drug from the PubChem database (PDB, https://www.rcsb.org/, accessed on 29 August 2026). The 3D structures of the target proteins, the receptors, were obtained from the Protein Data Bank (PDB, https://www.rcsb.org/) [21]. Autodock Vina is utilized for protein-ligand docking, while Pymol is employed to visualize molecular docking images. Given the three-dimensional (3D) structure of a protein and a ligand, can predict their binding sites and affinity for computer-aided drug discovery. A binding energy of less than −5 kcal/mol was defined as indicative of effective ligand-receptor binding, and the binding energy less than −7 kcal/mol indicated strong binding activity. The lower the binding energy, the stronger the resultant docking interaction. (Importantly, generative artificial intelligence (GenAI) has not been used in this paper.)

## 3. Results

### 3.1. MR Estimates and Sensitivity Analysis Findings of Genetically Predicted 51 Proteins on Dystonia

**Figure 2:**
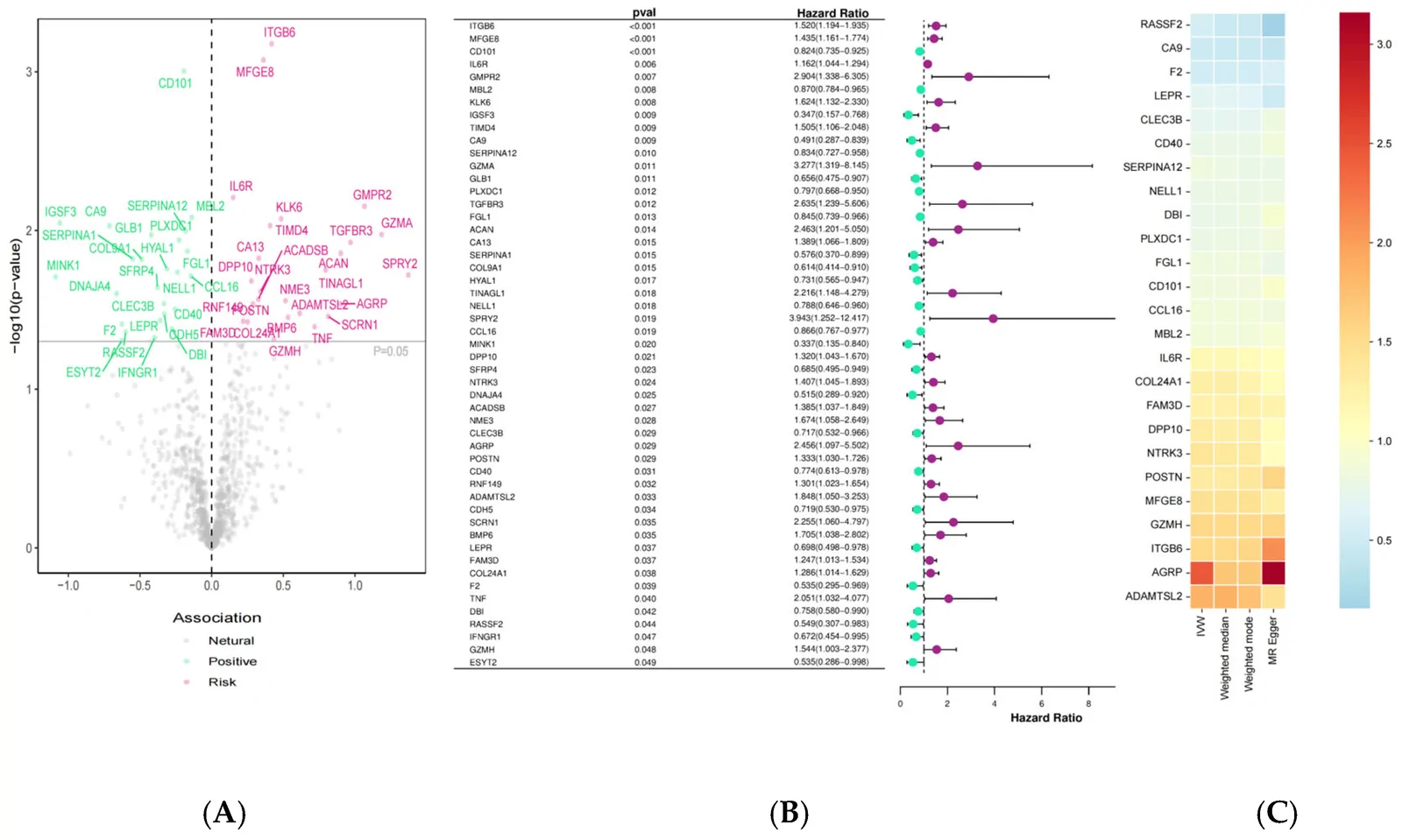
**Volcano plot and Forest plot of the MR results: Effects of 51 plasma proteins on dystonia.** (**A**): Volcano plot depicting the results of Mendelian randomization (MR) analysis, illustrating the associations between 51 proteins and dystonia risk, red represents proteins associated with an increased risk of dystonia (risk), and green represents proteins associated with a decreased risk of dystonia (positive). (**B**): Forest plot showing the MR-predicted associations between 51 proteins and dystonia risk, with significant associations (*p* < 0.05) based on IVW or Wald ratio results. (**C**): Among the 51 proteins identified by Mendelian randomization, 25 were analyzed using four methods (IVW, weighted median, weighted mode, and MR Egger). The results were visualized with heatmaps.

After strictly adhering to the filtering criteria for IVs in this study, a total of 941 plasma proteins were ultimately involved in the MR analysis, the results can be found in Supplementary Table S1. Relevant SNP information can be found in Supplementary Table S2. When the number of SNPs is fewer than three, the IVW method is applied. When there are three or more SNPs, four statistical methods are utilized for analysis (IVW, weighted median, weighted mode, and MR Egger). Notably, among the initial 2923 plasma-protein pQTLs, IVW-based MR analysis identified 51 proteins with nominally-significant genetically-predicted associations with dystonia risk (Supplementary Table S3, Figure 2A,B). After FDR correction, 51 proteins showed nominal association (*p* < 0.05, *q* > 0.05). The complete results are presented in Supplementary Table S3. Among them, 25 of proteins were statistically analyzed using 4 methods (Supplementary Table S4, Figure 2C). For the remaining 26 proteins, given that their number of SNPs was fewer than three, only the IVW method was employed. Per-standard-deviation (SD) increase in the genetically-predicted plasma protein level, the odds ratio (OR) for dystonia risk ranged from 0.337 (95% CI: 0.135–0.840) for misshapen-like kinase 1 (MINK1) to 3.943 (95% CI: 1.252–12.417) for sprouty homolog 2 (SPRY2). The corresponding OR was 2.051 (95% CI: 1.032–4.077) for tumor necrosis factor (TNF) (Figure 2B). In addition, considering the vulnerability of the IVW method to weak IVs bias, the implementation of multiple sensitivity analyses (Cochran Q test, MR-Egger intercept, MR-PRESSO, RadialMR, Causal analysis, Steiger test, and Leave-one-out analysis) substantiated the robustness and reliability of the MR estimates. Heterogeneity and horizontal-pleiotropy assessments were performed for the 25 proteins supported by ≥3 instrumental SNPs; Cochran’s Q, MR-Egger intercept, and MR-PRESSO analyses revealed no evidence of heterogeneity or horizontal pleiotropy among these proteins. It should be noted that 26 proteins supported by fewer than three SNPs were thus not subjected to heterogeneity or pleiotropy testing. Cochran’s Q test for SNP heterogeneity, MR-Egger intercept test for horizontal pleiotropy, and Steiger directionality test for causal orientation validation are presented in Supplementary Tables S5, S6 and S7, respectively.

### 3.2. Functional and Pathway Enrichment Analysis of Genetically Predicted 51 Proteins

**Figure 3:**
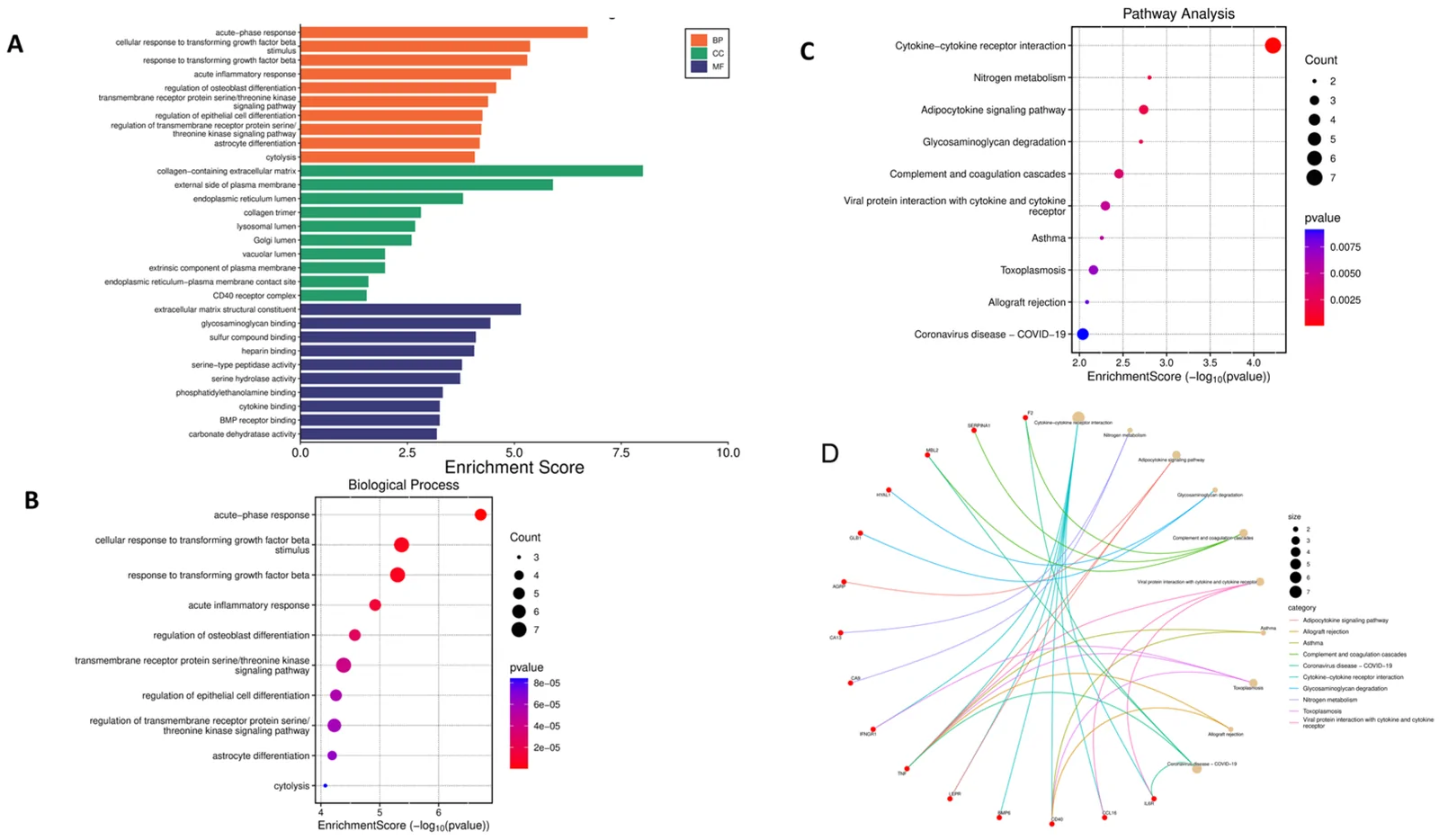
**GO and KEGG enrichment of dystonia-associated proteins.** (**A**): The top 10 terms in Biological Process (BP), Cellular Component (CC), and Molecular Function (MF) of GO enrichment are presented in the bar plot. (**B**): The top 10 terms in BP based on the enrichment score are shown in the bubble plot. (**C**): The top 10 terms in KEGG are depicted in the bubble plot. (**D**): The top 10 terms in KEGG are presented in the net plot.

The GO and KEGG enrichment analyses were conducted using 51 genetically predicted proteins identified through MR. We obtained 117 statistically significant (FDR < 0.05) GO function enrichment entries, including 90BP, 21MF, and 6 CC terms in total (Supplementary Table S8). The top 10 terms in BP, CC, and MF were selected and plotted in the column chart (Figure 3A). BP mainly involve the acute-phase response, cellular response to transforming growth factor beta stimulus, response to transforming growth factor beta, acute inflammatory response (Figure 3B). In the MF category, the target proteins were mainly involved in extracellular matrix structural constituent, glycosaminoglycan binding, sulfur compound binding. In the CC category, the target proteins were classified into collagen-containing extracellular matrix, external side of plasma membrane and endoplasmic reticulum lumen. The pathways with the highest enrichment levels included the cytokine-cytokine receptor interaction, nitrogen metabolism, adipocytokine signaling pathway, and glycosaminoglycan degradation (Supplementary Table S8). The top 10 potential signaling pathways were presented as bubble plots (Figure 3C). After performing the enrichment analysis with the clusterProfiler R package, a cnetplot be generated using the cnetplot function (Figure 3D). The results showed that genes were most enriched in the cytokine-cytokine receptor interaction signaling pathway.

### 3.3. Protein-Protein Interaction Network and Hub-Gene Network Analysis of Dystonia-Associated Proteins

**Figure 4:**
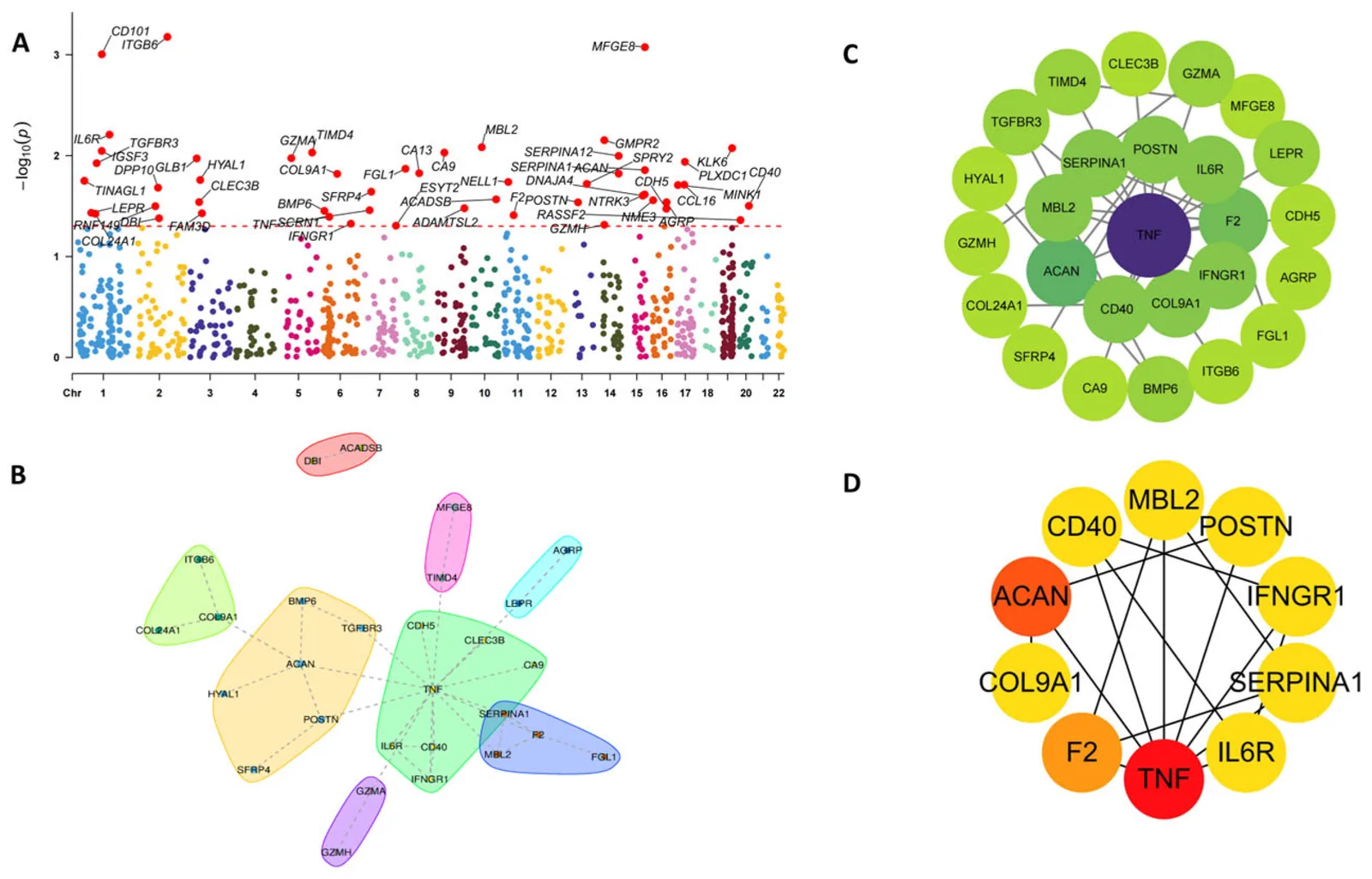
**Network prediction and function enrichment of dystonia.** (**A**): Manhattan plot of 51 dystonia-associated proteins. (**B**): Regulatory network among 51 dystonia-related proteins. (**C**): PPI network diagram of core targets. (**D**): Top 10 Hub-genes of core targets.

The Manhattan plot exhibited the distribution of genetic locus associated with 51 identified dystonia-associated proteins and significant genes were labelled in Figure 4A. Briefly, Manhattan plots revealed that the most significant genes, encompassing *CD101*, *ITGB6*, *IL6R*, *TNF*, *F2*, *ACAN*, etc. Among the 51 dystonia-associated proteins identified, we observed 21 community relationships within the regulatory network (Figure 4B). we applied the Cytoscape to establish and visualize a PPI network of dystonia-associated proteins (Figure 4C). Moreover, we used the cytoHubba plugin in Cytoscape to screen 10 hub-genes. TNF, F2 and ACAN were identified as key candidates among them. (Figure 4D).

### 3.4. Single-Cell RNA Annotations

**Figure 5:**
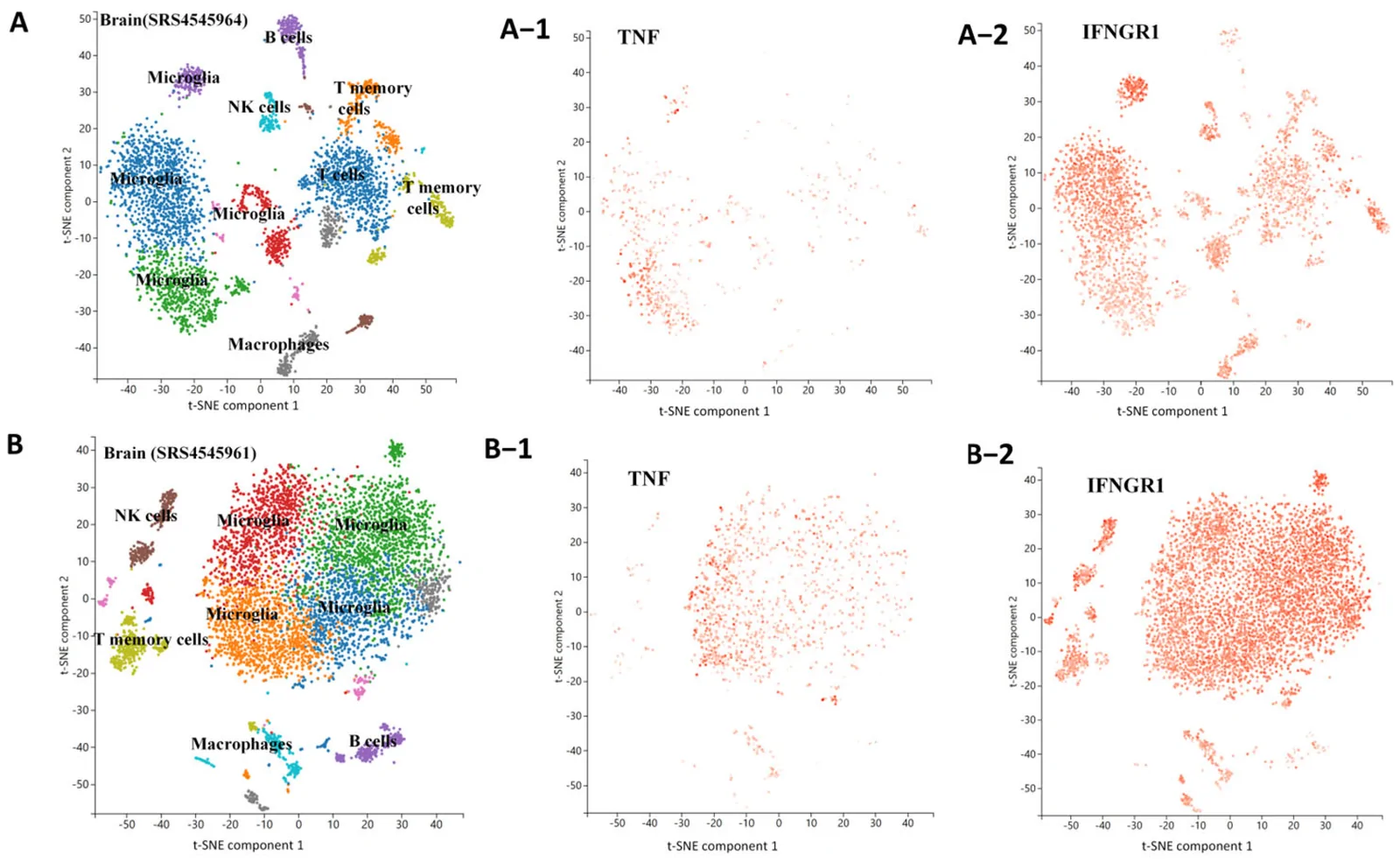
**Single-cell RNA annotations.** (**A**): Single-cell RNA annotation dataset for a brain cluster (SRA866994: SRS4545964). (**A-1**): High expression of the TNF gene in microglia cells of this cluster. (**A-2**): The IFNGR1 gene shows high brain tissue specificity and is highly expressed in all cell types of this cluster. (**B**): Another single-cell RNA annotation dataset for a brain cluster (SRA866994: SRS4545961). (**B-1**): High TNF gene expression in microglia cells of this cluster. (**B-2**): The IFNGR1 gene exhibits high brain tissue specificity and high expression across all cell types in this cluster.

We selected two datasets (SRA866994: SRS4545964; and SRA866994: SRS4545961) (Figure 5A,B) and analyzed the expression levels of 10 hub-genes in these datasets. The single-cell RNA annotations for brain cluster specificity revealed that the *TNF* gene was highly expressed in microglia cells (Figure 5(A-1,B-1)). The *IFNGR1* gene exhibited high tissue specificity in brain and highly expressed in cells such as microglia, macrophages, NK cells, T memory cells, and B cells (Figure 5(A-2,B-2)). The other genes, such as *F2*, *ACAN*, *MBL12*, *IL6R*, *CD40*, *POSTN*, *COL9A1*, and *SERPINA1*, exhibit either minimal expression or no expression at all. The expression levels of each gene were consistently observed across the two datasets, thereby validating the reliability of the results.

### 3.5. Drug Enrichment Analysis and Molecular Docking

**Figure 6:**
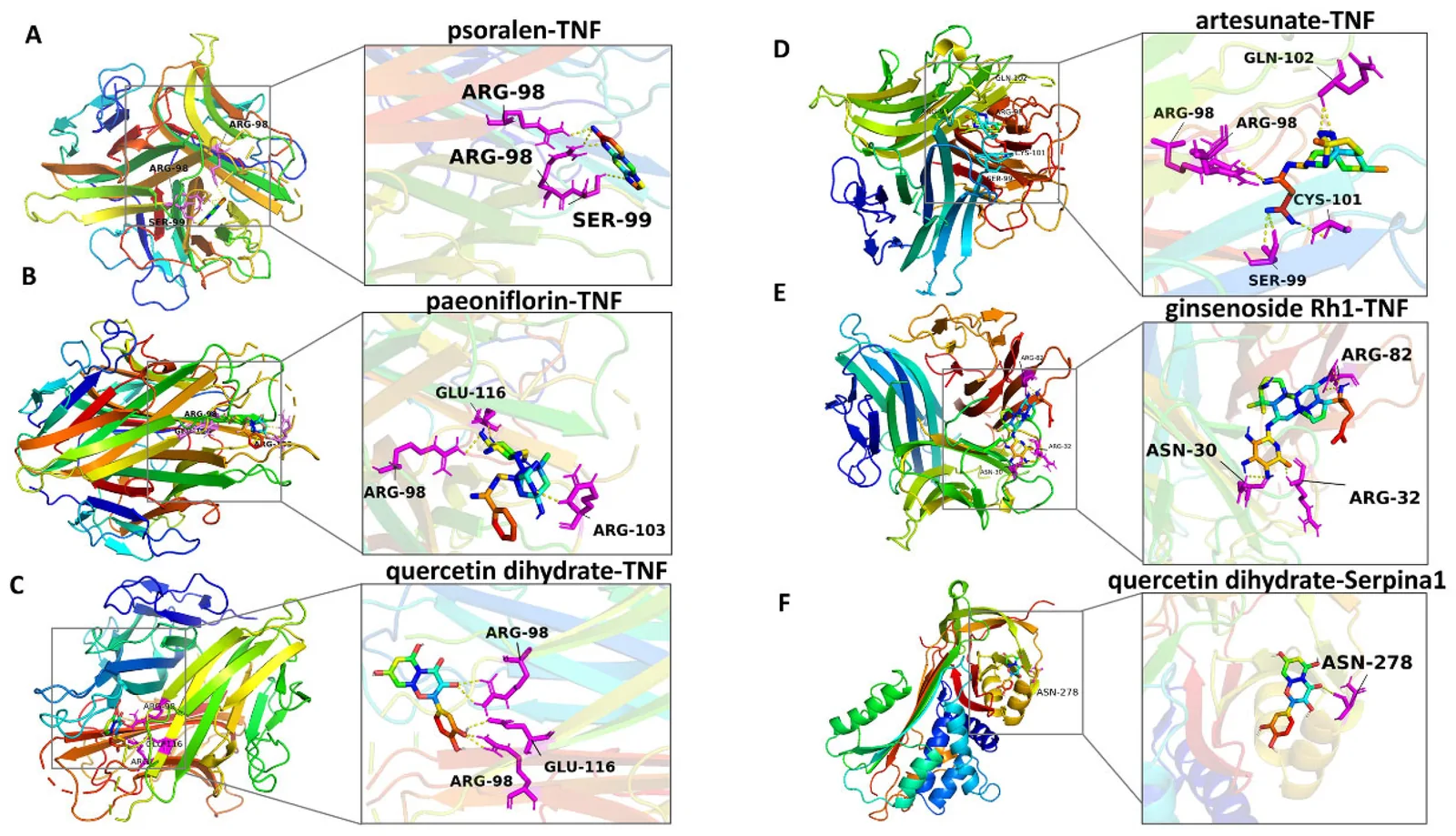
**Molecular docking.** (**A**): Binding mode of TNF and psoralen; (**B**): Binding mode of TNF and paeoniflorin; (**C**): Binding mode of TNF and quercetin dihydrate; (**D**): Binding mode of TNF and artesunate; (**E**): Binding mode of TNF and ginsenoside Rh1; (**F**): Binding mode of Serpina1 and quercetin dihydrate.

Firstly, drug enrichment analysis targeting all drugs and 10 hub-genes were performed, we identified 617 drugs with significant enrichment (FDR < 0.05), including five monomers derived from TCM (paeoniflorin, artesunate, ginsenoside Rh1, psoralen, and quercetin dihydrate) (Supplementary Table S9). The key TCM monomer and protein were validated by molecular docking utilizing the binding energy that reached or fell below −5.0 kcal/mol as the criterion [22]. Subsequently, molecular docking was performed between these five monomers and their respective targets (TNF, Serpina1) (Figure 6A–F), followed by an analysis of their binding energy. The binding energies obtained in our molecular-docking simulations ranged from −6.7 to −9.1 kcal/mol, indicating that these small-molecule monomers exhibited favorable binding affinity for the target protein. Paeoniflorin-TNF, quercetin dihydrate-TNF, and artesunate-TNF yielded the lowest binding energy (−9.1 kcal/mol), indicating stronger binding affinity. In comparison, ginsenoside Rh1-TNF (−6.8 kcal/mol), psoralen-TNF (−6.7 kcal/mol), and quercetin dihydrate-SERPINA1 (−6.7 kcal/mol) exhibited weaker predicted binding interactions.

## 4. Discussion

This study represents the pioneering utilization of MR analysis, and single-cell RNA, and network pharmacology to explore potential therapeutic targets for dystonia. Using MR analysis, we identified nominally-significant associations between genetically-predicted levels of 51 proteins and dystonia risk (IVW, *p* < 0.05, *q* > 0.05 after FDR correction). As we know, MR analysis can be influenced by reverse causality [23]. Therefore, Steiger filtering analysis was performed to establish the directionality of causal effects. Additionally, we specifically selected cis-pQTL as an IVs. We further identified proteins that have the same variants as dystonia and effectively eliminated any potential deviations. GO and KEGG enrichment analyses revealed that these proteins are involved cellular response to transforming growth factor-β stimulation and cytokine-cytokine receptor interaction, which potentially involved the regulation of the transmembrane receptor protein serine/threonine kinase signaling pathway. PPI network analysis identified 10 hub-genes (*TNF*, *ACAN*, *F2*, *IFNGR1*, etc.) forming critical functional modules within the 51-protein interactome. The single-cell RNA annotations for brain cluster specificity revealed microglial-specific TNF overexpression, suggesting CNS-specific inflammatory regulation. Computational drug screening prioritized five phytochemicals from TCM, with paeoniflorin, artesunate and quercetin dihydrate showing superior TNF binding affinity (−9.1 kcal/mol) in molecular docking simulations. This multi-omics integration provides an integrative framework for targeting neuroinflammatory pathways, particularly highlighting microglial TNF signaling as a druggable mechanism in dystonia pathogenesis.

Mendelian randomization studies for movement disorders have largely prioritized Parkinson’s disease and essential tremor, with limited MR research targeting dystonia itself. One recent study combined FAERS pharmacovigilance data with proteome-wide MR and colocalization to explore molecular mechanisms of drug-induced dystonia, focusing on medication-related adverse events but without cell-type annotation or therapeutic drug screening. Our study differs by investigating general dystonia susceptibility: we integrated proteome-wide MR, single-cell RNA-seq, network pharmacology and molecular docking to identify hub susceptibility genes (e.g., TNF) enriched in brain microglia and prioritize candidate therapeutic monomers. This multi-omics workflow connects causal proteomic findings to potential drug discovery, representing a key advancement compared with previously published MR analyses for dystonia [24].

Of the 10 hub-genes derived from the PPI network, only TNF and SERPINA1 were prioritized for molecular docking analysis owing to their close biological links with neuroinflammation in movement disorder pathogenesis. Other hub-genes were excluded from the present docking screening and reserved for further investigation. The neuroinflammatory pathological process in the central nervous system (CNS) is mainly driven by the abnormal activation of its resident immune cells, microglia and astrocytes [25]. As a core regulatory hub, tumor necrosis factor (TNF) has a double-edged sword characteristic in the nervous system: it promotes M1-type microglial polarization through the TNFR1 signaling pathway, and activates regulatory T cells via the TNFR2 pathway to maintain immune homeostasis. Mechanism studies have shown that TNF is secreted by injury-responsive macrophages, activated microglia, and infiltrating CD4+ T lymphocytes, and regulates the neuroinflammatory process [26]. Importantly, TNF triggers a pro-inflammatory cytokine cascade by activating the NF-κB signaling pathway, forming a self-amplifying inflammatory cycle [27,28]. This chronic inflammatory microenvironment leads to neuronal calcium homeostasis imbalance and synaptic plasticity damage, which is directly related to cognitive and motor impairments in neurodegenerative diseases [29,30]. GWAS have revealed that dystonia patients carry microglia-specific inflammatory gene variations, confirming that neuroinflammation is the core pathological mechanism [31,32]. Animal experiments have demonstrated that the toxin 3-Nitropropionic acid (3-NP) induces neuropathology in the mouse striatum and substantia nigra, including excitotoxicity, neuroinflammation, and extensive neuronal atrophy. These pathologies are characterized by dystonia, closely resembling those observed in human patients [33].

The analysis of plasma protein-wide association studies integrates pQTLs and dystonia GWAS data to explore the causal relationship between plasma proteins and the risk of dystonia. The study’s notable strengths include the utilization of the MR methodology to minimize latent confounders and reverse causality bias, followed by the incorporation of cis-pQTLs to enhance the level of proof (cis-pQTL > trans-pQTL > eQTL). TNF-α is one of the major mediators of the neuroinflammation associated with neurodegeneration [34]. Paeoniflorin (PF), the primary active compound in the Chinese herb Paeoniae alba Radix, has been shown to alleviate neuroinflammation and dopaminergic neurodegeneration in Parkinson’s disease models [35]. PF inhibits microglial activation in the hippocampus and downregulates the expression of the pro-inflammatory cytokine TNF-α, thereby exhibiting both anti-inflammatory and neuroprotective effects [36,37,38,39,40,41,42,43]. Recent studies have highlighted the potential protective role of artemisinin, a well-known antimalarial drug, in various neurological disorders [44,45]. Artesunate inhibits the production of proinflammatory mediators in LPS-stimulated BV-2 microglial cells [46]. Our research further suggests that paeoniflorin, artesunate and quercetin dihydrate may target TNF and serve as potential therapeutic TCM monomers for dystonia, with the possibility of alleviating neuroinflammation. TNF remains a biologically plausible candidate given its central role in neuroinflammation, microglia-specific expression, and prior literature. The molecular docking results are presented as exploratory.

An important consideration in drug target prioritization is the alignment between the genetically predicted effect direction and the intended therapeutic mechanism. Our MR analysis indicated that higher genetically predicted TNF levels are associated with increased dystonia risk. Therefore, the therapeutic hypothesis is that TNF inhibition should reduce dystonia risk or ameliorate disease progression. Among the three TCM monomers prioritized here—paeoniflorin, artesunate, and quercetin—all have been reported to suppress TNF expression or signaling in microglial or inflammation models, with strong multi-study support. The alignment between MR directionality and predicted pharmacology strengthens the biological plausibility of these candidates, though direct evidence in dystonia models remains lacking.

However, there are certain limitations in this study. Firstly, the inclusion of only participants of European origin may impact the generalizability of the MR results. Secondly, while this study applied MR approach to screen for causal associations between proteins and dystonia, a genetics-led drug target prioritization method (Priority index, PI) was not employed in this study for identifying under-explored targets. A key limitation is the modest statistical power of our proteome-wide MR analysis. Our proteome-wide MR analysis did not identify any protein that remained significant after Benjamini-Hochberg FDR correction (*q* < 0.05). Therefore, all reported protein-disease associations should be considered preliminary and require independent replication in larger cohorts or functional validation. We acknowledge that our focus on TCM monomers is not exhaustive. It should be emphasized that our molecular docking analysis was performed primarily as an exploratory in-silico screening to yield preliminary evidence for potential interactions between candidate compounds and TNF. This computational docking study was not designed for quantitative comparisons of binding affinity or target selectivity against clinically available anti-TNF therapeutics. Accordingly, the reported binding energies (−6.7 to −9.1 kcal/mol) were only used to rank the predicted binding capacity of the screened compounds, and we refrain from drawing any conclusions regarding their superiority over marketed drugs. Synthetic small-molecule TNF inhibitors and approved anti-TNF biologics also have therapeutic potential for TNF-driven neuroinflammation. However, biologics face substantial CNS delivery challenges, and synthetic TNF inhibitors have not yet been optimized for BBB penetration. Future comparative studies evaluating TCM monomers alongside synthetic and biologic TNF inhibitors in dystonia models are warranted. Additionally, suitable animal models are currently unavailable, practical constraints prevented us from conducting animal experiments; however, we plan to incorporate experiments into our program or collaborate with other labs in order to enhance knowledge in this field. We eagerly anticipate delving deeper into these drug targets and their potential therapeutic approaches for movement disorders in future studies.

## 5. Conclusions

Our study identified nominally significant, genetically-predicted associations between 51 proteins and dystonia risk. Specifically, molecular-docking analysis suggested that TCM-derived monomers including paeoniflorin, quercetin dihydrate, and artesunate may interact with the TNF protein. These compounds are promising candidate agents that require further experimental validation, which may provide new clues for developing therapeutic targets for dystonia. Our findings highlight the potential of TCM monomers as candidate targeted interventions for dystonia.

## Data Availability

UKB-PPP (protein) Sun et al. External, opens in a new tab. https://metabolomips.org/ukbbpgwas/, (accessed on 29 August 2026). The original contributions presented in the study are included in the article/Supplementary Material. Further inquiries can be directed to the corresponding authors.

## Supplementary Materials

The following supporting information can be downloaded at: https://www.mdpi.com/article/10.3390/genes17091067/s1, Table S1: List of 941 plasma-protein traits included in the Mendelian randomiza tion analysis; Table S2: Detailed information on single-nucleotide polymorphism instrumental variables used for MR analyses; Table S3: Primary MR results for plasma proteins associated with dystonia risk; Table S4: MR results estimated by four analytical methods for proteins with ≥3 instrumental SNPs; Table S5: Cochran’s-Q test results for heterogeneity assessment; Table S6: MR-Egger intercept test results for horizontal pleiotropy evaluation; Table S7: Steiger directionality test results for causal-effect orientation validation; Table S8: GO and KEGG functional enrichment results of 51 candidate proteins associated with dystonia risk; Table S9: Drug enrichment analysis results based on candidate hub-genes.

## Abbreviations

The following abbreviations are used in this manuscript:

MR	Mendelian randomization
GWAS	Genome-wide association studies
SNPs	single nucleotide polymorphisms
pQTLs	protein quantitative trait loci
IVs	instrumental variables
TCM	traditional Chinese medicine
NP	Network pharmacology
LD	linkage disequilibrium
IVW	inverse-variance weighting
OR	Odds ratios
CAUSE	Causal Analysis Using Summary Effect estimates
MR-PRESSO	MR Pleiotropy Residual Sum and Outlier
GO	Gene Ontology
BP	Biological Processes
CC	Cellular Components
MF	Molecular Functions
PPI	Protein-Protein Interaction
scRNA-seq	single-cell RNA-sequencing
3D	three-dimensional
CNS	central nervous system
TNF	tumor necrosis factor
3-NP	3-Nitropropionic acid
PF	Paeoniflorin
FDR	False Discovery Rate
PDB	Protein Data Bank
MINK1	misshapen-like kinase 1 protein
SPRY2	protein sprouty homolog 2
